# 1-((2,4-Dichlorophenethyl)Amino)-3-Phenoxypropan-2-ol Kills *Pseudomonas aeruginosa* through Extensive Membrane Damage

**DOI:** 10.3389/fmicb.2018.00129

**Published:** 2018-02-08

**Authors:** Valerie Defraine, Veerle Liebens, Evelien Loos, Toon Swings, Bram Weytjens, Carolina Fierro, Kathleen Marchal, Liam Sharkey, Alex J. O’Neill, Romu Corbau, Arnaud Marchand, Patrick Chaltin, Maarten Fauvart, Jan Michiels

**Affiliations:** ^1^Centre of Microbial and Plant Genetics, KU Leuven, Leuven, Belgium; ^2^Center for Microbiology, Vlaams Instituut voor Biotechnologie, Leuven, Belgium; ^3^Data Integration and Biological Networks, Ghent University, Ghent, Belgium; ^4^School of Molecular and Cellular Biology, University of Leeds, Leeds, United Kingdom; ^5^CISTIM Leuven vzw, Leuven, Belgium; ^6^Centre for Drug Design and Discovery, Leuven, Belgium; ^7^Smart Electronics Unit, Department of Life Sciences and Imaging, imec, Leuven, Belgium

**Keywords:** *Pseudomonas aeruginosa*, mechanism of action studies, membrane damage, antibiotic tolerance, anti-persister therapies

## Abstract

The ever increasing multidrug-resistance of clinically important pathogens and the lack of novel antibiotics have resulted in a true antibiotic crisis where many antibiotics are no longer effective. Further complicating the treatment of bacterial infections are antibiotic-tolerant persister cells. Besides being responsible for the recalcitrant nature of chronic infections, persister cells greatly contribute to the observed antibiotic tolerance in biofilms and even facilitate the emergence of antibiotic resistance. Evidently, eradication of these persister cells could greatly improve patient outcomes and targeting persistence may provide an alternative approach in combatting chronic infections. We recently characterized 1-((2,4-dichlorophenethyl)amino)-3-phenoxypropan-2-ol (SPI009), a novel anti-persister molecule capable of directly killing persisters from both Gram-negative and Gram-positive pathogens. SPI009 potentiates antibiotic activity in several *in vitro* and *in vivo* infection models and possesses promising anti-biofilm activity. Strikingly, SPI009 restores antibiotic sensitivity even in resistant strains. In this study, we investigated the mode of action of this novel compound using several parallel approaches. Genetic analyses and a macromolecular synthesis assays suggest that SPI009 acts by causing extensive membrane damage. This hypothesis was confirmed by liposome leakage assay and membrane permeability studies, demonstrating that SPI009 rapidly impairs the bacterial outer and inner membranes. Evaluation of SPI009-resistant mutants, which only could be generated under severe selection pressure, suggested a possible role for the MexCD-OprJ efflux pump. Overall, our results demonstrate the extensive membrane-damaging activity of SPI009 and confirm its clinical potential in the development of novel anti-persister therapies.

## Introduction

Modifying existing antibiotic scaffolds upon emergence of resistance has proven a successful strategy to extend a drug class’ utility in the past. However, recent data suggest that multidrug-resistance increases at an alarming rate while few novel antibacterials reach the market (Wright, 2014; O’Neill, 2015). A particular issue are multidrug-resistant Gram-negative pathogens such as *Pseudomonas aeruginosa*, posing additional challenges to antibiotic discovery due to their highly impermeable outer membrane (Livermore, 2002; Breidenstein et al., 2011), and the so-called ESKAPE pathogens (*Enterococcus faecium*, *Staphylococcus aureus*, *Klebsiella pneumoniae*, *Acinetobacter baumannii*, *P. aeruginosa*, and *Enterobacter* spp.), efficiently evading antibiotic treatment and responsible for the majority of bacterial infections (Rice, 2008; Delcour, 2009; Poole, 2011). Since no new antibiotic scaffolds active against Gram-negative pathogens have been identified in the last decades, physicians are reverting to the use of polymyxins, once avoided due to toxic effects, as a last-resort treatment for these strains. Therefore, new antibacterial scaffolds are desperately needed (Falagas and Kasiakou, 2005; Walsh and Wencewicz, 2014).

**FIGURE 1:**
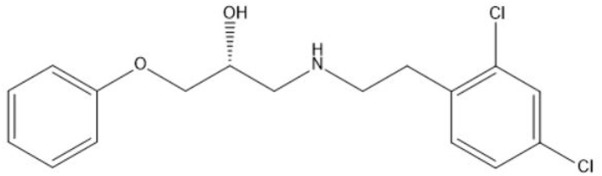
Chemical structure of the propanol-amine derivative 1-((2,4-dichlorophenethyl)amino)-3-phenoxypropan-2-ol (SPI009).

Contributing to the difficult treatment of bacterial infections is the presence of persister cells which constitute a small but important fraction of phenotypic variants tolerant to treatment with high doses of antibiotics (Lewis, 2010). Their occurrence in many bacterial pathogens combined with a demonstrated link between persistence and the recalcitrant nature of chronic bacterial infections renders persisters a serious threat to immunocompromised patients and effective anti-persister treatments are much needed (Fauvart et al., 2011; Zhang, 2014; Fisher et al., 2017). Previous research revealed the strong antibacterial effect of the novel anti-persister molecule SPI009 (Liebens et al., 2017; **Figure 1**) as an adjuvant in combination therapies against different bacterial pathogens. In addition, the compound proved highly successful in the treatment of intracellular and *in vivo P. aeruginosa* infections when combined with the fluoroquinolone ciprofloxacin. SPI009 sensitizes bacteria to antibiotic activity and, strikingly, restores antibiotic sensitivity even in resistant strains. In addition, SPI009 monotherapy exhibited extensive inhibition and eradication activity in biofilms of *P. aeruginosa* and *S. aureus* (Defraine et al., 2017). The current need for novel antibacterials active against Gram-negative species, together with the unique characteristic of SPI009 to kill both normal and persister cells, prompted us to further investigate the mode of action of this compound.

Current approaches to identify the next generation of antibacterials involve high-throughput screenings of natural and chemical products, the characterization and adaptation of new antibacterial structures (Samanta et al., 2013; Mandal et al., 2017), genome hunting, whole-cell-based assays and the targeting of non-multiplying bacteria (Coates et al., 2002). As these options offer interesting alternatives that can bypass the identification of novel antibiotic targets, mechanism of action studies become increasingly important to characterize and select interesting candidates after initial discovery (Terstappen et al., 2007).

In this study, we set out to determine the mode of action of a recently discovered antibacterial compound, SPI009, showing a broad spectrum antibacterial effect and capable of killing both dividing cells and non-dividing or dormant persister cells (Liebens et al., 2017). While generating useful information for the further development of this compound as an antibacterial therapy, determination of the mode of action could also greatly assist in the development of future anti-persister therapies. A combination of genetic and cellular approaches was employed, revealing the membrane damaging activity of SPI009 and suggesting the ability of SPI009 to attack the bacterial membrane(s) both from the cytoplasm and extracellular environment.

## Materials and Methods

### Bacterial Strains, Media, and Growth Conditions

Bacterial strains were cultured in 1:20 diluted trypticase soy broth (1:20 TSB) at 37°C, shaking at 200 rpm. For solid medium, TSB was supplemented with 1.5% agar. The following antibacterials were used: ofloxacin, ciprofloxacin, erythromycin, polymyxin B, rifampicin (Sigma – Aldrich), fosfomycin, meropenem (TCI Europe), triclosan (Merck Chemicals), melittin (Bachem), and SPI009 (**Figure 1**). Concentrations are indicated throughout the text. Bacterial strains used in this study are listed in **Table 1**.

**Table 1:** Strains used in this study.

Strain	Description	Source or reference
***Pseudomonas aeruginosa***		
PA14 WT	Wild type UCBPP–PA14	Pierre Cornelis; Lee et al., 2006
YM WT	PAO1 WT, K767	De Kievit et al., 2001
YM64	*mexAB-oprM::FRT*, *mexXY::FRT*, *mexCD-oprJ::FRT*, *mexEF-oprN::FRT*	Morita et al., 2001
K1521	K767 Δ*mexCD-oprJ*	De Kievit et al., 2001
K1525	K767 Δ*mexXY*	De Kievit et al., 2001
Liberati WT	Wild type UCBPP-PA14 (Lib WT)	Liberati et al., 2006
Δ*galU*	PA14_38350::Mar2xT7 (52640)	Liberati et al., 2006
Δ*nfxB*	PA14_60860::Mar2xT7 (55219)	Liberati et al., 2006
ΔPA14_0812	PA14_08120::Mar2xT7 (47659)	Liberati et al., 2006
***Acinetobacter baumannii***		
Ab-84	MDR clinical isolate	García-Quintanilla et al., 2014
Ab-84R	Ab-84::40nt insertion at nt 321 of *lpxC*	García-Quintanilla et al., 2014

### Screening of a *P. aeruginosa* Mutant Library

A genetic screen of the *P. aeruginosa* PA14 transposon mutant library (Liberati et al., 2006) was performed to identify single gene knockouts sensitive or resistant for SPI009. Stationary phase mutant cultures were split in two and treated for 5 h with either 10 μg/mL ofloxacin or the combination of ofloxacin and 51 μg/mL of SPI009. Treated cultures were diluted 1:100 in fresh TSB medium and incubated at 37°C, shaking at 200 rpm. Growth was monitored over a total period of 40 h by means of periodic OD595 measurements. Average OD595 was calculated for each 96-well plate and used to correct mutant OD595 values. Mutants were identified as sensitive if the OD595 after 24 h of growth was ≤0.3× average OD595 after 24 h. Alternatively, mutants having an OD595 >3× average were defined as resistant. The screening was performed twice to prevent false-positive hits and, to allow identification of SPI009 specific effects, selected mutants showing a clear sensitivity or resistance for ofloxacin were excluded. Resistant hits were additionally confirmed via detailed monitoring of growth in the presence of 51 μg/mL SPI009, using an automated OD reader (Bioscreen C). Functional enrichment analysis was performed based on PseudoCAP classifications and using Fisher’s exact test. A schematic overview of the described workflow can be found in Supplementary Figure S1.

### RNA Sequencing and Data Analysis

Overnight cultures of *P. aeruginosa* were diluted 1:100 in fresh 1/20 TSB medium and allowed to grow until late-exponential phase (OD595 = 0.2). Cells were treated for 15 min with 50 μM SPI009, 50 μM of the inactive analog SPI014 or 1% DMSO. Total RNA isolation was performed in triplicate for each sample, as previously described (Liebens et al., 2014). The Ribo-Zero^TM^ rRNA Removal Kit for Gram-negative bacteria (Epicentre) was used to deplete ribosomal RNA and RNA samples were sent to the Genomics Core facility of EMBL (Heidelberg, Germany). The quality of the raw sequencing reads was verified using FastQC after which genomic alignments of the reads were performed with Bowtie2, using the *P. aeruginosa* UCBPP-PA14 genome as a reference (NC_008463.1). Differential expression analysis between treated and control samples was done using the DESeq2 package with a False Discovery Rate threshold of 5% (Love et al., 2014). Genes with a log_2_ fold-change above 1 and no differential expression under the inactive compound treatment were selected, allowing the detection of SPI009 specific effects on gene expression. Functional annotation of the obtained results was performed using PseudoCAP functional classes obtained from www.pseudomonas.com (Winsor et al., 2016) and functional enrichment was assessed using Fisher’s exact test. A UCBPP-PA14 interaction network was created using the STRING database (Szklarczyk et al., 2011) where only reactions with a minimum reliability score of 0.8 were retained. PheNetic was run using both this network and the obtained omics data to generate a downstream interaction network, using the standard parameters and a cost of 0.25 (De Maeyer et al., 2013). Obtained networks containing more than two genes were visualized using Cytoscape (Shannon et al., 2003).

### Macromolecular Synthesis Assay

An overnight culture of *P. aeruginosa* PA14 wild type (WT) was diluted 1:10 in 1/20 TSB and grown to an OD600 of 0.3 at 37°C (late exponential phase). Radiolabeled precursors for DNA (1 μCi/mL [methyl-3H]-thymidine), RNA (2.5 μCi/mL [5,6-3H]-uridine), protein (2.5 μCi/mL L-[4,5-3H]-leucine), peptidoglycan (2.5 μCi/mL D-[6-3H(N)]-glucosamine hydrochloride), and fatty acids (1 μCi/mL [2-3H]-glycerol) were added after which cultures were treated with 17 μg/mL SPI009 or 8× MIC concentrations of relevant control antibiotics. Thirty minutes after onset of treatment, 100 μL samples were added to 3.5 mL of ice-cold 10% TCA and precipitates were collected under vacuum on 25 mm glass microfiber filters (Whatman^®^ Grade GF/C). Filters were washed twice with 4 mL ice-cold distilled water and added to 3.5 mL scintillation liquid (Ultima-Flo M, PerkinElmer). Incorporation of the different radiolabels was assessed using a Hidex 300SL scintillation counter. Counts per minute at different treatment conditions were used to evaluate the incorporation of radiolabeled precursors relative to the untreated control, as previously described (Cotsonas King and Wu, 2009; Grzegorzewicz et al., 2012; Nowakowska et al., 2013; Ling et al., 2015).

### Fluorescein Leakage Assay

Small unilamellar vesicles (SUVs) representing the Gram-negative membrane and loaded with carboxyfluorescein (CF) were produced as described previously (Randall et al., 2013; Gerits et al., 2016). The total phospholipid concentration was kept at 25 μM, containing a mixture of 1,2-dioleoyl-sn-glycero-3-phosphoethanolamine (DOPE)/1,2-dioleoyl-sn-glycero-3-phospho-(1′-rac-glycerol) (DOPG) (4:1) (Avanti Polar Lipids, Inc). Liposomes were treated with increasing concentrations of SPI009 and an inactive chemical analog, SPI023, keeping the final DMSO concentration at 1% (v:v). Release of CF (λex = 485 nm, λem = 520 nm) was measured in function of time. The percentage of CF leakage was determined relative to the treatment with 0.5% Triton X-100.

### Assessment of Inner and Outer Membrane Permeabilization

Inner membrane permeabilization was examined using a SYTOX Green uptake assay, as previously described (Gerits et al., 2016). A PA14 WT culture was grown until late exponential phase and corrected to a final OD595 of 0.5 in 1× phosphate-buffered saline supplemented with 1 μM SYTOX Green. Cultures were treated with Milli-Q (MQ; untreated control), DMSO (1%; carrier control), 10 μg/mL melittin (1× MIC) and increasing concentrations of SPI009 and transferred to the wells of a black microtiter plate (clear bottom). Fluorescence (λex = 504 nm, λem = 523) and absorbance (OD595) were measured every minute, using a Synergy MX multimode reader (BioTek) at 37°C.

*Pseudomonas aeruginosa* outer membrane permeabilization by SPI009 was measured using a 1-*N*-phenylnaphthylamine (NPN, Sigma, United States) uptake assay (Gerits et al., 2016). Briefly, a *P. aeruginosa* PA14 WT culture was grown until late exponential phase, after which the OD595 was corrected to 0.5 in 5 mM HEPES (pH = 7.2). A total of 150 μL volumes of culture were treated with MQ (untreated control), DMSO (1%; carrier control), 0.625 μg/mL polymyxin B (1× MIC) and different concentrations of SPI009 (4.25–34 μg/mL) and transferred to the wells of a black microtiter plate (clear bottom). Fifty microliters of a 40 μM NPN solution in 5 mM HEPES (pH = 7.2) was added and fluorescence was measured immediately using a Synergy MX multimode reader (BioTek) at 37°C.

Independent assays for outer and inner membrane permeabilization assessment were performed three times. Measured fluorescence signals were divided by well-specific OD595 to correct for cell density after which the values of the respective untreated controls were subtracted. Results are expressed in relative fluorescence units.

### Microscopic Confirmation of Membrane Damage

Overnight cultures of *P. aeruginosa* and *S. aureus* were treated for 20 min with 0.5% DMSO (carrier control) or 34 μg/mL SPI009, centrifuged and stained with 10 μg/mL *N*-(3-triethylammoniumpropyl)-4-(6-(4-(diethyl amino)phenyl)hexatrienyl)pyridinium dibromide (FM^®^4-64, Molecular Probes). Samples were spotted on 2% agarose pads for imaging with Zeiss Axio imager Z1 fluorescence microscope, using an EC Plan-NEOFLUAR 100× objective (λex = 540–580 nm; λem = 593–668 nm).

### Interaction of SPI009 with LPS

To assess possible interaction between SPI009 and the lipid A compound of Gram-negative LPS layers, a whole-cell BODIPY^TM^ TR Cadaverine displacement assay was performed. Briefly, a late-exponential PA14 WT culture was corrected to an OD595 of 0.3 in 50 mM Tris–HCl and added to the BODIPY^TM^ TR Cadaverine conjugate (BC, 5 μM; Life Technologies) in the wells of a black microtiter plate (clear bottom). Cultures were incubated for 2 h to allow BC binding after which equimolar amounts of Tris–HCl (negative control), meropenem, polymyxin B and SPI009 were added and fluorescence (λex = 580 nm, λem = 620 nm) was measured continuously for 1 h. Fluorescence values from the negative control (Tris–HCl) were used to correct for background fluorescence.

### Antibacterial Assay

The effect of SPI009 on different bacterial cultures was assessed as described previously (Liebens et al., 2017). Briefly, stationary phase cultures were treated for 5 h with DMSO (carrier control) and different concentrations of SPI009. After treatment, cultures were washed twice in 10 mM MgSO_4_ and appropriate dilutions were plated onto solid agar plates to assess the number of colony forming units.

### Generation and Whole Genome Sequencing of SPI009-Resistant Mutants

In an attempt to generate resistant mutants, *P. aeruginosa* was plated out on solid TSB agar plates containing high concentrations of SPI009. The plates were incubated at 37°C for a total of 10 days but no colonies were able to grow, proving the absence of any resistance development under the specific conditions (our own, unpublished data). Alternatively, resistance development was assessed using a MIC-based protocol as previously described, with minor modifications (Briers et al., 2014; Ling et al., 2015). An initial MIC test was performed in three independent *P. aeruginosa* PA14 WT cultures with ofloxacin and SPI009, according to EUCAST standards (EUCAST, 2003). After 24 h of growth at 37°C, shaking, the MIC value was determined as the minimal concentration that completely inhibited bacterial growth. A new MIC assay was prepared using 1:100 diluted cells at MIC/4 as a starting condition. The assay was repeated for 10 passages with daily assessment and, if necessary, adjustment of antibiotic and compound concentrations. Intermediate and endpoint cultures were stored at -80°C in glycerol (25% v/v) for further analysis. Genomic DNA of the *P. aeruginosa* PA14 WT strain and the three evolved resistant mutants was isolated from overnight cultures grown in 1/20 TSB using the DNeasy Blood & Tissue Kit (Qiagen) following the manufacturer’s instructions. DNA quantity and purity were verified using a NanoDrop ND-1000, after which samples were sent to the Genomics Core Facility of EMBL (Heidelberg, Germany) for whole genome sequencing on the Illumina HiSeq 2500 platform. Assembly of the 125 bp paired-end reads and further analysis was performed using CLC Genomics Workbench v8.0. Genome sequences of the resistant mutants were aligned with the genome of the PA14 WT strain in order to detect genetic differences, taking into account a coverage above 10× and cutoff frequency of 75%. Identified non-synonymous mutations were confirmed via PCR amplification and Sanger sequencing (GATC Biotech).

## Results

### Genetic Analysis of the SPI009 Mode of Action

**FIGURE 2:**
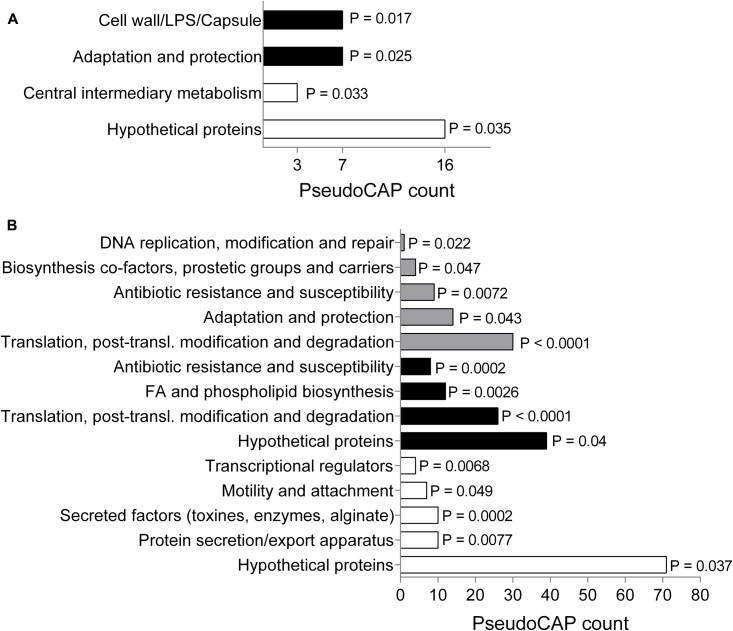
Genetic analysis of SPI009 mode of action. **(A)** Functional enrichment of the obtained set of sensitive (black bars) and resistant (white bars) mutants using PseudoCAP classifications. *P*-values were determined using a two-tailed Fisher’s exact test (α = 0.05). PseudoCAP counts represent the number of mutants in the respective classes. **(B)** Functional enrichment analysis of the up- and downregulated differentially expressed genes (log_2_ fold change >1) as determined after RNA sequencing upon treatment with SPI009. A two-tailed Fisher’s exact test was used to determine *P*-values for the classes of downregulated genes (white bars), upregulated genes (black bars), and the total set (gray bars).

To gain more information about the mode of action of SPI009 and allow the detection of possible persister-specific effects, individual single-gene knockouts from a *P. aeruginosa* mutant library were treated with ofloxacin alone or in combination with SPI009 and screened for altered sensitivity to SPI009 (see overview in Supplementary Figure S1). Analysis of the obtained screening results revealed a total of 118 and 37 different mutants that showed an increased or decreased sensitivity for SPI009, respectively. Functional enrichment analysis of the sensitive mutants based on their PseudoCAP functions (Winsor et al., 2016), revealed an over-representation for genes involved in “adaptation and protection” and “cell wall/LPS/capsule” (**Figure 2A** and Supplementary Table S1). The relatively low levels of resistance, combined with the functional enrichment analysis (**Figure 2A**) suggest that single-gene knockouts are not sufficient to obtain significant resistance toward the anti-persister effects of SPI009 and point to a more general effect of the compound.

Next, we compared genome-wide gene expression levels of *P. aeruginosa* PA14 WT following treatment with either SPI009 or an inactive analog. RNA sequencing analysis generated a list of 297 genes that were specifically differentially expressed (log_2_-fold change >1) upon treatment with SPI009 (Supplementary Table S2). Functional enrichment analysis of up- and downregulated genes (**Figure 2B**), combined with network analysis (Supplementary Figure S2) revealed a first group of SPI009 upregulated genes to be involved in antibacterial efflux and multidrug resistance, suggesting the increased efforts of the cell to protect itself against SPI009. Another group of mostly upregulated genes are involved in fatty acid metabolism and degradation, suggesting altered amounts of available fatty acids upon treatment of the cell with SPI009. Furthermore, there is a downregulation of multiple genes involved in virulence; including phenazine biosynthesis, pilus assembly and protein secretion and the bacterial Type VI secretion system and biofilm formation (Imperi et al., 2013).

Taking both genetic analyses into account, there does not appear to be a single process or pathway that emerges as the target for SPI009. Instead, membrane-related functions are perturbed, supplemented with more general effects in different regulatory and metabolic pathways. This could point to SPI009 causing membrane damage.

### SPI009 Inhibits Macromolecular Biosynthesis in a Non-specific Manner

**FIGURE 3:**
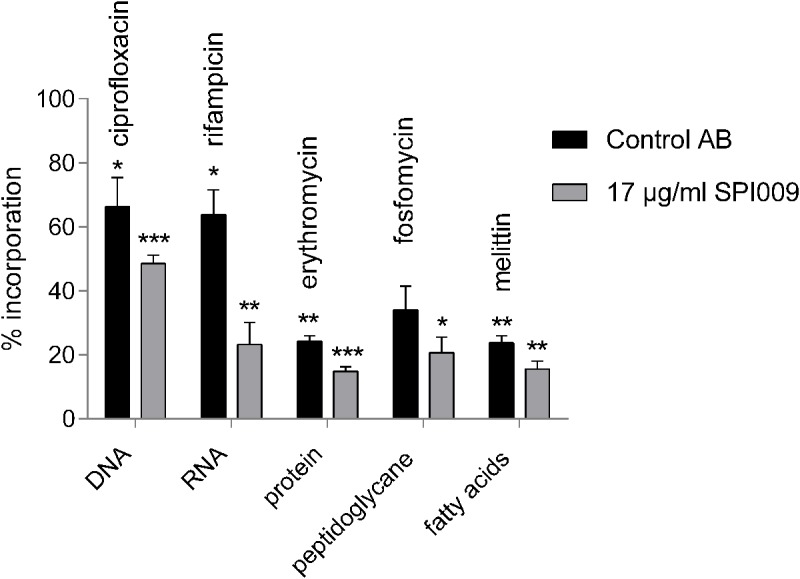
SPI009 inhibits macromolecular synthesis. Incorporation of [methyl-^3^H]-thymidine (DNA), [5,6-^3^H]-uridine (RNA), L-(4,5-^3^H)-leucine (protein), D-[6-^3^H(N)]-glucosamine (peptidoglycan), 2-^3^H-glycerol (fatty acids) by *P. aeruginosa* PA14 WT after treatment with relevant control antibiotics at 8× MIC concentrations or 17 μg/mL of SPI009. Incorporation was measured after 30 min and expressed relative to an untreated control. Bars represent the average of at least three independent repeats ± SEM. Statistical analysis was performed on “counts per minute” obtained upon radioactivity detection after treatment of the bacterial cells with MQ (untreated control), the control antibiotic or 17 μg/mL SPI009. One-way ANOVA (α = 0.05) with appropriate correction for multiple testing was used to determine statistically relevant differences between the untreated control and antibiotic or untreated control and SPI009 treatment. ^∗^*P* < 0.05; ^∗∗^*P* < 0.01; ^∗∗∗^*P* < 0.001.

To further explore the hypothesis of SPI009-induced membrane damage and rule out other bacterial mechanisms targeted by the compound, a macromolecular synthesis assay was performed (Cotsonas King and Wu, 2009). Addition of 17 μg/mL of SPI009 strongly reduced incorporation of radio-labeled precursors for DNA, RNA, proteins, fatty acids, and peptidoglycan, resulting in a more than 50% decrease in synthesis for all macromolecules tested (**Figure 3**). When compared to different antibiotics, known to inhibit incorporation of precursors, 1/3× MIC concentrations of SPI009 show a generally stronger inhibitory effect, a pattern previously reported for membrane-damaging compounds (Hobbs et al., 2008; Nowakowska et al., 2013; Masschelein et al., 2015; Gerits et al., 2016).

### SPI009 Is Capable of Disrupting Artificial Bacterial Bilayers

**FIGURE 4:**
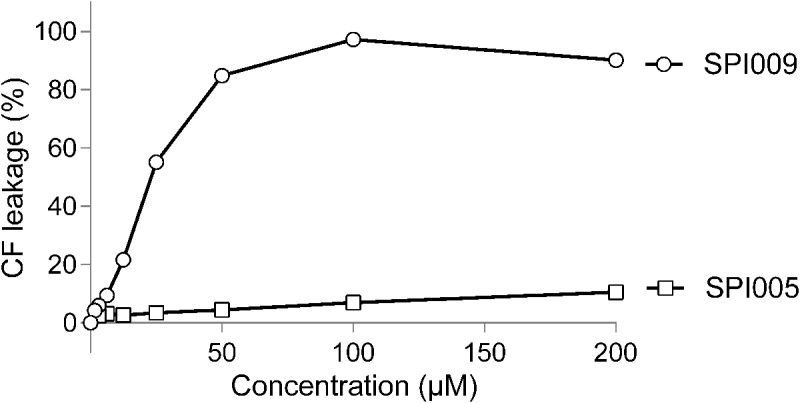
SPI009 causes extensive CF leakage. SUVs were treated for 15 min with increasing concentrations (*x*-axis) of SPI009 (open circles) or the inactive analog SPI005 (open squares). Increasing concentrations of ofloxacin, used as a negative control, did not result in any CF leakage (data not shown). % CF leakage was determined by fluorescence measurements, corrected for background fluorescence and expressed relative to the positive control (0.5% Triton X-100). Data points represent the mean of three independent repeats ± SEM.

To further confirm the suggested membrane damaging effect of SPI009, its capacity to disrupt lipid bilayers that mimic the Gram-negative inner membrane was tested. Increasing concentrations of SPI009 clearly induced CF leakage in a concentration-dependent manner, while the inactive analog SPI005, displaying no antibacterial or anti-persister effect, and the conventional antibiotic ofloxacin, did not cause any significant leakage (**Figure 4**). When comparing SPI009 with the membrane damaging antibiotic polymyxin B, 50% CF leakage was obtained at concentrations of 11.92 ± 0.07 and 1.16 ± 0.04 μg/mL, representing 0.185× MIC and 1.85× MIC concentrations of SPI009 and polymyxin B (Supplementary Figure S3), respectively. These results indicate that SPI009 is indeed capable of effectively disturbing an artificial lipid bilayer and further support the membrane-damaging hypothesis.

### Membrane Permeabilization Studies

**FIGURE 5:**
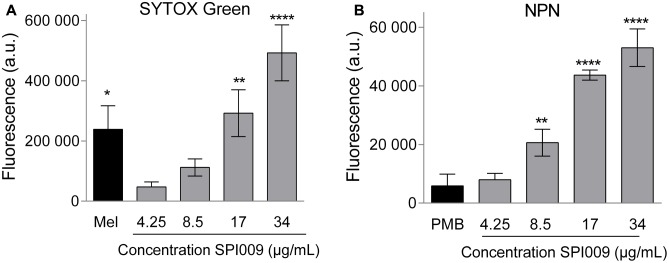
SPI009 extensively permeabilizes both inner and outer membrane of *P. aeruginosa.* **(A)** Effect of increasing concentrations of SPI009 on the inner membrane permeability, as measured by the SYTOX Green uptake assay. Cells were treated for 30 min using melittin (Mel; 1× MIC) as a positive control. **(B)** Outer membrane permeability after treatment with increasing concentrations of SPI009, using polymyxin B (PMB, 1× MIC) as a positive control. Data for both assays represent the mean of at least three independent repeats ± SEM. Statistical comparisons with the untreated control were performed using a one-way ANOVA (α = 0.05) with Dunnett’s correction for multiple comparison (^∗^*P* < 0.05, ^∗∗^*P* < 0.01, ^∗∗∗∗^*P* < 0.0001).

To evaluate membrane disruption activity of SPI009 on whole *P. aeruginosa* cells, NPN and SYTOX Green assays were carried out, allowing the investigation of respectively outer and inner membrane permeabilization. SYTOX Green shows a strong increase in fluorescence upon binding to DNA. This is, however, only possible when the inner membrane of the cell is compromised, thus correlating the observed fluorescence with the amount of inner membrane damage (Roth et al., 1997). Thirty-minute treatment of *P. aeruginosa* with increasing concentrations of SPI009 caused a strong increase in fluorescence as compared to the untreated control (**Figure 5A** and Supplementary Figure S4A). At concentrations of 17 μg/mL (= 0.33× MIC), the observed membrane damage was comparable to the effect of treatment with 1× MIC concentrations of melittin, the active compound in bee venom known to induce inner membrane damage (Raghuraman and Chattopadhyay, 2007).

Next, permeabilization of the outer membrane was assessed by means of the hydrophobic fluorescent probe NPN. Bacterial cells normally exclude NPN. Consequently, increasing fluorescence caused by the insertion of the probe in the phospholipid bilayer is indicative of damage to the bacterial outer membrane (Helander and Mattila-Sandholm, 2000). Fluorescence measurements revealed a rapid outer membrane permeabilization by SPI009 in a clear concentration-dependent manner (**Figure 5B** and Supplementary Figure S4B). In comparison, treatment with 1× MIC concentration of polymyxin B resulted in a comparable fluorescence level at 1/6× MIC concentrations of SPI009. These results strongly support the hypothesis that SPI009 is capable of disrupting the bacterial membrane, and this for both the inner and outer membrane of *P. aeruginosa*.

### Microscopic Confirmation of Membrane Damage

**FIGURE 6:**
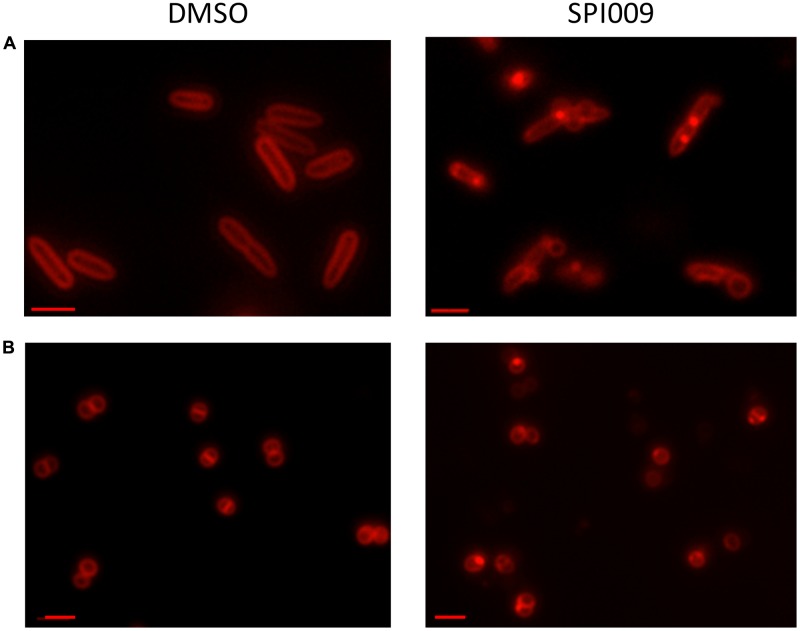
Microscopic confirmation of SPI009 induced membrane damage. Overnight cultures of **(A)** *P. aeruginosa* and **(B)** *S. aureus* cultures were treated for 20 min with 1% DMSO (carrier control) or 34 μg/mL of SPI009. Treated cells were stained with 10 μg/mL of FM^®^ 4-64 and visualized using a Zeiss Axio imager Z1 fluorescence microscope equipped with an EC Plan-NEOFLUAR 100× objective (λ_ex_ = 540–580 nm; λ_em_ = 593–668 nm). Scale bars correspond to 2 μM. Pictures are representatives of repeated experiments.

Since the bacterial membrane is such a critical part of the cell’s architecture, membrane stains are commonly used for microscopic visualization of cell integrity. Treatment of *P. aeruginosa* and *S. aureus* with DMSO (1%, carrier control) resulted in uniformly stained membranes while addition of 34 μg/mL SPI009 induced brightly fluorescent membrane accumulations (**Figure 6**). In the Gram-negative *P. aeruginosa*, a second phenotype was visible: stained membrane blebs, possibly originating from severe outer membrane deformations (Kulp and Kuehn, 2010). The observed membrane accumulations after treatment with SPI009 confirm a direct effect of the compound on the bacterial membrane for both Gram-negative and Gram-positive species.

### SPI009 Interacts with the Lipid A Compound of the Bacterial LPS Layer

**FIGURE 7:**
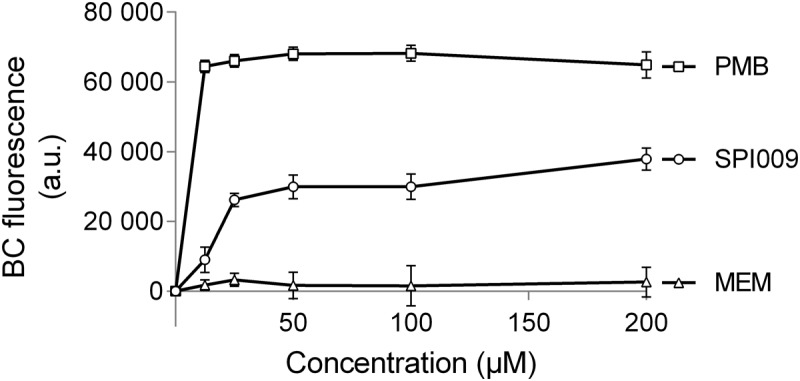
SPI009 interacts with the LPS layer. BC bound *P. aeruginosa* cultures were treated with equimolar amounts of SPI009, polymyxin B (positive control; PMB) and meropenem (negative control; MEM). Fluorescence was measured continuously, using λ_ex_ = 580 nm and λ_em_ = 620 nm, and corrected for background fluorescence. Results represent the average of four independent repeats ± SEM with BC fluorescence values obtained after 1 h.

The BODIPY^TM^-TR-cadaverine probe (BC) was used to reveal possible interactions of SPI009 with the bacterial LPS layer. If compounds are added that have the ability to bind lipid A, BODIPY^TM^-TR Cadaverine will be displaced, resulting in a strong increase in fluorescence (Torrent et al., 2008; Gerits et al., 2016). Positive and negative controls consisted of, respectively, polymyxin B, known to use the interaction with lipid A for self-promoted uptake and resulting in cell lysis (Yu et al., 2015), and meropenem, not capable of interacting with LPS. Comparison of the effects of equimolar amounts of SPI009 and these controls demonstrated a clear time and concentration-dependent interaction between SPI009 and lipid A (**Figure 7**). However, since it was previously shown that SPI009 maintains its antibacterial and anti-persister activity in Gram-positive bacteria, the interaction with lipid A in the bacterial LPS layer cannot be the sole mechanism of SPI009-induced membrane damage.

### The Role of Efflux Pumps in SPI009 Activity

**FIGURE 8:**
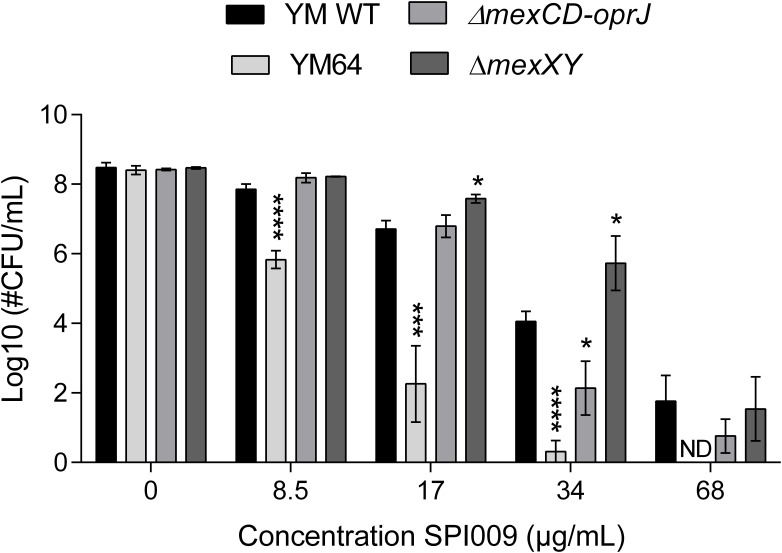
Activity of SPI009 in efflux mutants. *P. aeruginosa* efflux mutants YM64, Δ*mexCD-oprJ* and Δ*mexXY*, together with their WT strain YM, were treated for 5 h with increasing concentrations of SPI009. Significant differences in sensitivity toward SPI009 between the WT and different mutants were detected using multiple *t*-tests (α = 0.05) with Holm–Sidak correction for multiple comparisons and represented by ^∗^*P* < 0.05; ^∗∗∗^*P* ≤ 0.001; and ^∗∗∗∗^*P* < 0.0001. Values represent the average of at least three independent repeats with error bars depicting SEM values. ND, not detected.

The SPI009-induced inner membrane damage, as indicated by the SYTOX Green uptake assay, could be a secondary effect resulting from extensive outer membrane damage caused by SPI009. Alternatively, the compound may enter the bacterial cell and cause membrane damage from within. To explore these possibilities, different efflux mutants were evaluated for their sensitivity toward SPI009 (**Figure 8** and Supplementary Figure S5). Upon treatment with SPI009, the PAO1-derived YM64 mutant, lacking the four major mex operons of *P. aeruginosa*; *mexAB-oprM*, *mexCD-oprJ*, *mexEF-oprN*, and *mexXY-oprM* (Morita et al., 2001), showed a significantly decreased survival for all concentrations tested. Treatment with 8.5, 17, and 34 μg/mL SPI009 caused significant 2.0 ± 0.3, 4.5 ± 0.9, and 3.7 ± 0.4 log unit decreases in survival as compared to the YM WT, respectively, while 68 μg/mL of SPI009 was capable of completely eradicating the efflux mutant. To further explore the role of the different efflux pumps missing in YM64, separate Δ*mexCD-oprJ* and Δ*mexXY* mutants were also analyzed. For these, only the Δ*mexCD-oprJ* strain showed decreased survival compared to the YM WT after treatment with 34 μg/mL SPI009. The obtained results clearly show that some *P. aeruginosa* efflux pumps, including MexCD-OprJ, are capable of actively removing SPI009 from the bacterial cell and suggest the involvement of other Mex pumps, most likely not MexXY-OprM. These experiments confirm the inner membrane as an important target of SPI009, in addition to the observed outer membrane permeabilization.

### Role of Natural Membrane Permeability in SPI009 Sensitivity

**FIGURE 9:**
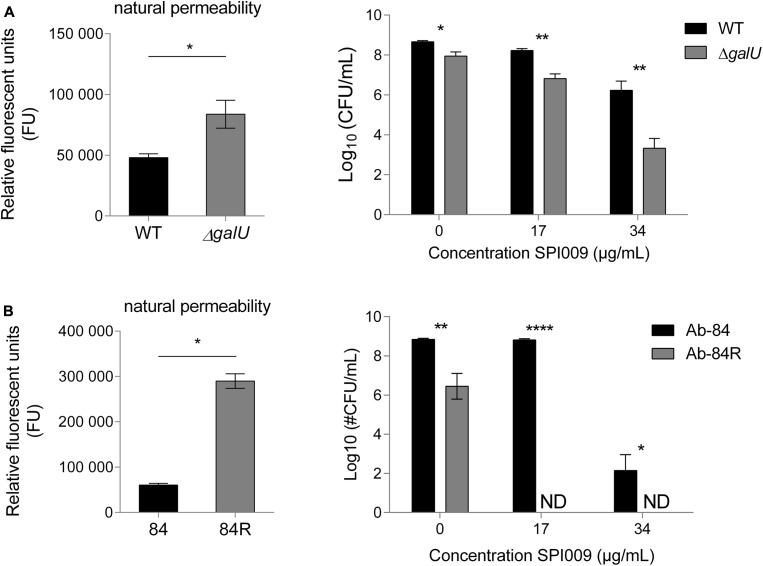
Cells displaying increased membrane permeability show higher sensitivity toward SPI009. Mutants resulting in LPS biosynthesis malfunction or complete loss of the LPS layer were selected for **(A)** *P. aeruginosa* and **(B)** *Acinetobacter baumannii*, respectively. Natural membrane permeability was assessed by means of NPN assays of untreated cultures (left panel). Statistical analysis was done by means of a single unpaired, two-tailed *t*-test (α = 0.05). Next, stationary phase cultures of WT and mutant strains were treated for 5 h with increasing concentrations of SPI009 (right panel). Statistical analysis was done by means of multiple *t*-tests (α = 0.05) to compare the antibacterial effect of SPI009 between the WT and mutant strain, with Holm–Sidak correction for multiple testing. Displayed data points show the average of at least three independent repeats ± SEM. ^∗^*P* < 0.05; ^∗∗^*P* < 0.01; ^∗∗∗^*P* < 0.001; and ^∗∗∗∗^*P* ≤ 0.0001; ND, not detected.

Since the natural membrane permeability of different bacterial species contributes to their intrinsic antibiotic resistance (Breidenstein et al., 2011), we investigated whether this also affected the activity of SPI009. *P. aeruginosa* Δ*galU*, is no longer capable of synthesizing UDP-glucose, a precursor required for the formation of the glycosyl residues found in the bacterial LPS layer (Choudhury et al., 2005). Natural membrane permeability was assessed by measuring NPN fluorescence of MQ-treated samples and revealed a significantly higher permeability for Δ*galU* (**Figure 9A**). Treatment with 17 or 34 μg/mL SPI009 revealed respective 1.4 ± 0.3 log and 2.9 ± 0.7 log unit decreases in survival as compared to the WT strain.

We next wanted to evaluate the effect of SPI009 in the complete absence of LPS. However, no *P. aeruginosa* strain lacking LPS has been described to date. In contrast, a 40 nt insertion in *A. baumannii lpxC* results in the complete loss of the bacterial LPS layer without affecting viability (García-Quintanilla et al., 2014). Moreover, like *P. aeruginosa*, *A. baumannii* is also a member of the Pseudomonadales and an important contributor to the spread of antibiotic resistance. As compared to the WT strain Ab-84, the absence of the LPS layer greatly increased the observed natural membrane permeability (**Figure 9B**). The differential sensitivity of these strains for SPI009 shows a more pronounced character than for *P. aeruginosa* since treatment with 17 μg/mL of SPI009 already completely eradicated the LPS-deficient Ab-84R strain. Together, these results indicate that the structure of the bacterial LPS layer, which partly determines membrane integrity and strength, strongly influences the susceptibility of the cell toward SPI009.

### Generation and Analysis of SPI009-Resistant Mutants

Being so far unable to generate spontaneous SPI009-resistant mutants on solid growth medium (our own, unpublished data), an evolution experiment using the MIC broth dilution method was used to generate mutants showing a 10-fold increase in the SPI009 MIC. MIC values slowly increased for the strains grown in the presence of SPI009 while resistance to the conventional antibiotic ofloxacin showed a more abrupt transition with a first plateau of 16-fold increase in MIC being reached after just 2 days (Supplementary Figure S6A). The results obtained for ofloxacin are in agreement with previous fluoroquinolone resistance evolution experiments (Wong et al., 2012; Briers et al., 2014; Ling et al., 2015). Decreased sensitivity for SPI009 of the evolved strains was confirmed via MIC and plate assays, where treatment with 68 μg/mL of SPI009 caused a maximal 1.46 log decrease in the number of surviving cells (Supplementary Figure S6B). Whole genome sequencing and analysis of the evolved strains revealed two identical non-synonymous SNPs in each of the parallel lines. A first mutation involved a 47A > C change in PA14_08120 (PA0625), a phage tail length determination protein located in the outer membrane or outer membrane vesicles (Choi et al., 2011; Winsor et al., 2016). PA0625 is part of a 16-ORF gene cluster coding for R-type phage tail-like pyocins in *P. aeruginosa*, bacteria-produced bacteriocins that are capable of depolarizing the cytoplasmic membrane in sensitive cells and inhibiting active transport (Nakayama et al., 2000; Ghequire and De Mot, 2014; Choudhary et al., 2015). A second SNP, 88G > A, was identified in nfxB, the negative regulator of the MexCD-OprJ efflux system. Interestingly, this SNP is located in a predicted helix-turn-helix region responsible for DNA-binding. Other similar mutations in this region have been shown to disturb the binding to the MexCD-OprJ promotor and thus cause overexpression of this efflux pump and active expulsion of SPI009 from the cell (Okazaki and Hirai, 1992; Purssell and Poole, 2013).

A transposon mutant of nfxB showed a significant decrease in sensitivity toward SPI009 resulting in 1.7 ± 0.4 and 3.1 ± 0.9 log unit increases in survival as compared to the WT after treatment with 17 and 34 μg/mL of SPI009, respectively (Supplementary Figure S6B). In contrast to the whole genome sequencing results, full gene knockout of PA14_08120 did not cause a significant increase in survival. Taken together, these results confirm the hypothesis that the MexCD-OprJ efflux is capable of protecting the cell against SPI009-mediated membrane damage. Further research will, however, be necessary to unravel the exact role of PA14_08120 in this mechanism.

## Discussion

Increased understanding of persister formation mechanisms and the general acknowledgment of their clinical importance has resulted in a growing number of reports on anti-persister molecules, contributing to potential future treatment options in the fight against bacterial infections (Wood, 2015; Van den Bergh et al., 2017). Targeting persisters is likely to greatly improve patient outcomes but unfortunately the rational target-based design of anti-persister therapies remains a great challenge. Contributing to this are the limited numbers of persister cells, the incomplete knowledge and redundancy in mechanisms controlling persister formation and the observation that these processes are often species-specific (Harms et al., 2016; Michiels et al., 2016; Van den Bergh et al., 2017)^.^ A possible way of bypassing these issues is the use of well-designed whole-cell screenings that can identify novel compounds based on anti-persister activity rather than target (Coates et al., 2002). We recently reported the use of such a screening in the identification of SPI009 (1-((2,4-dichlorophenethyl)amino)-3-phenoxypropan-2-ol) (Liebens et al., 2017), a small molecule capable of directly killing persister cells of clinically relevant Gram-negative and Gram-positive pathogens in different *in vitro* and *in vivo* set-ups. Other anti-persister compounds reported to directly kill bacterial persister cells use varying strategies such as depolarization and destruction of the cell membrane, DNA cross-linking, inhibition of essential enzymes, and generation of reactive oxygen species (Helaine and Kugelberg, 2014; Wood, 2015; Van den Bergh et al., 2017). Several characteristics of SPI009, such as its broad-spectrum activity, ability to tackle both dividing and non-dividing cells and the potentiation of mechanistically different antibiotics presented a first indication of a non-specific target. Identifying the mechanism of action for this novel anti-persister and antibacterial compound is not only important for the further development of possible therapies but also increases our knowledge about persister cells and contributes to the identification of possible targets for future anti-persister therapies (Terstappen et al., 2007).

In this study, we present the detailed exploration of the mode of action of SPI009, combining complementary genetic and cellular approaches. Several lines of evidence support that SPI009 kills persister and non-persister cells by causing extensive membrane damage. The overall inhibition of macromolecular synthesis at relatively low concentrations of SPI009, together with the obtained genetic data, provided us with the indication that SPI009 induced membrane damage (Hobbs et al., 2008; Nowakowska et al., 2013; Masschelein et al., 2015; Gerits et al., 2016). Several membrane and whole-cell-based assays confirmed this hypothesis and revealed the possibility of SPI009 to efficiently and extensively damage both the outer and inner bacterial membrane. Furthermore, microscopic analysis revealed membrane damage and severe outer membrane deformations and blebs in *P. aeruginosa*. The observed changes upon treatment of the Gram-positive *S. aureus* suggest a similar mechanism of membrane damage, but further research will be necessary to confirm this. Since the integrity of the bacterial membrane remains crucial for the viability of persister cells, membranes have previously been suggested as potential targets for anti-persister strategies (Hurdle et al., 2011). Several anti-persister compounds described in literature, such as the Artilysin^®^ Art-175 (Briers et al., 2014; Defraine et al., 2016), membrane-acting peptides (Chen et al., 2011), HT61 (Hu et al., 2010; Hubbard et al., 2017), and AM-0016 (Zou et al., 2013) use this strategy to efficiently tackle antibiotic-tolerant persister cells of both Gram-negative and Gram-positive species.

Although SPI009 proved capable of interacting with the lipid A moiety of the bacterial LPS layer, the demonstrated activity in Gram-positive species and LPS-deficient strains exclude LPS as the primary binding target of SPI009. However, the architecture of the LPS layer and resulting membrane permeability do have a strong influence on SPI009 activity. Possible explanations include the physical barrier formed by the LPS sugars, its influence on overall membrane strength or the changes in membrane charge due to the absence or presence of sugars and phosphate groups (Rana et al., 1991; Papo and Shai, 2005). Additionally, the increased SPI009 sensitivity of the *P. aeruginosa* YM64 mutant, lacking the four major Mex efflux pumps (Morita et al., 2001) and resulting in increased intracellular concentrations of the compound, provided evidence that SPI009 can cause cytoplasmic membrane damage and suggests the possible use of efflux pump inhibitors to further increase SPI009 activity.

Additional experiments revealed a possible role for MexCD-OprJ in the efflux of SPI009, but the substantial difference in sensitivity between YM64 and Δ*mexCD-oprJ* suggests that additional efflux mechanisms are involved. Interestingly, both the genetic screen and RNA sequencing revealed genes belonging to the *mexCD*-*oprJ* system and its regulator *nfxB*, previously reported to be inducible by membrane-damaging agents and best known for its role in fluoroquinolone resistance (Morita, 2003; Fraud et al., 2008; Purssell and Poole, 2013). Besides increasing the efflux of antibacterial compounds through MexCD-OprJ, Δ*nfxB* also influences cell membrane permeability (Okazaki and Hirai, 1992), thus suggesting an alternative role for *nfxB* in SPI009 resistance. The involvement of this bacterial efflux pump was corroborated through genetic analysis of the evolved SPI009-resistant strains, where all three independent mutants showed SNPs in *nfxB* and PA14_08120. While inactivation of *nfxB* strongly decreased sensitivity toward SPI009, this was not the case for PA14_08120. The R2 region of *P. aeruginosa* has previously been linked with antibiotic resistance. Fluoroquinolone induced production and release of pyocins was shown to cause cell lysis, while deletion of several R2 genes induced significant resistance to ciprofloxacin (Brazas and Hancock, 2005; Breidenstein et al., 2008). However, the lack of upregulation of any of the R2 genes in response to SPI009 (see Supplementary Table S2), suggests a different role for PA14_08120 in SPI009 susceptibility. Differences in survival between the *nfxB* knockout mutant and the SPI009-resistant strains could suggest the enhancement of the observed SPI009 resistance after accumulation of both mutations. The absence of SPI009 resistant mutants in earlier attempts, combined with the lack of any clear resistance phenotypes after single-gene knockout, predicted fitness defects of Δ*nfxB* (Stickland et al., 2010) and possibility of using efflux pump inhibitors (Lomovskaya and Watkins, 2001) all decrease the chances of SPI009 resistance emerging in *in vivo* situations.

Overall, the use of different combined approaches resulted in compelling evidence that the novel antibacterial compound SPI009 is capable of directly killing *P. aeruginosa* cells as a consequence of severe membrane damage. Further experiments revealed the ability of the compound to impair both the outer and inner membrane of *P. aeruginosa*, the latter being a direct consequence of SPI009 entry into the cell. The crucial importance of membrane integrity for survival of both active and metabolically inactive bacterial cells combined with the suggested limited resistance potential of membrane-damaging compounds (Hurdle et al., 2011) and the previously reported limited cytotoxicity (Liebens et al., 2017), further support the clinical potential of SPI009 and its role as a scaffold in the development of future anti-persister therapies.

## Author Contributions

Conceptualization: VD, VL, RC, AM, PC, MF, and JM; Methodology: VD, VL, MF, and JM; Formal analysis: VD and VL; Investigation: VD, VL, EL, TS, LS, BW, CF, and KM; Writing- original draft: VD; Writing- Review and Editing: VD, VL, AO, MF, and JM; Visualization: VD; Supervision: MF and JM.

## Conflict of Interest Statement

The authors declare that the research was conducted in the absence of any commercial or financial relationships that could be construed as a potential conflict of interest.
