# Supplementary material for: 1-((2,4-Dichlorophenethyl)Amino)-3-Phenoxypropan-2-ol Kills Pseudomonas aeruginosa through Extensive Membrane Damage
Source: Front Microbiol. 2018 Feb 8;9:129. doi: 10.3389/fmicb.2018.00129 (PMC5809444; doi:10.3389/fmicb.2018.00129)
Supplement: Supplementary file 1 [file Data_Sheet_1.pdf]

Supplementary Material

1-((2,4-dichlorophenethyl)amino)-3-phenoxypropan-2-ol kills *Pseudomonas aeruginosa* through extensive membrane damage

Valerie Defraine, Veerle Liebens, Evelien Loos, Toon Swings, Bram Weytjens, Carolina Fierro, Kathleen Marchal, Liam Sharkey, Alex J. O'Neill, Romu Corbau, Arnaud Marchand, Patrick Chaltin, Maarten Fauvart, Jan Michiels*

*** Correspondence:** Jan Michiels; jan.michiels@kuleuven.vib.be

1 Supplementary Data

1.1 Supplementary Figures

Figure S1

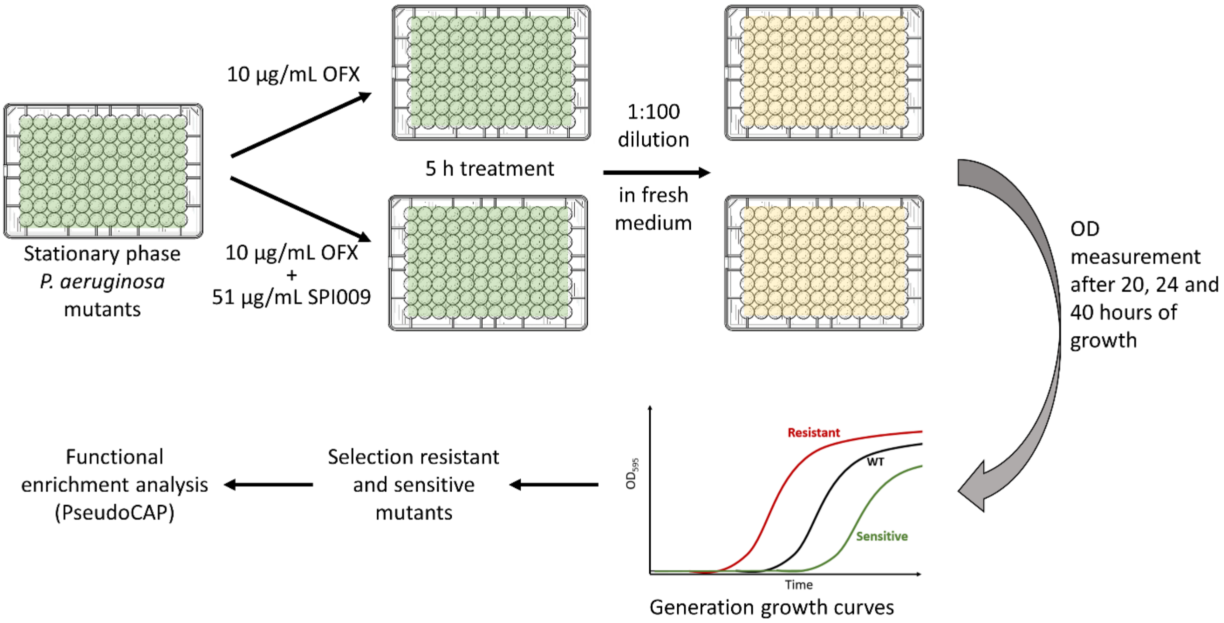

Figure S1: Schematic overview of the workflow employed for screening of the *P. aeruginosa* mutant library. *P. aeruginosa* mutants were inoculated in 1:20 TSB medium in 96-well microtiterplates. Overnight cultures were diluted 1:100 and grown to stationary phase after which the culture was split in two. One half was treated with 10 µg/mL ofloxacin while the second half was treated with the combination of 10 µg/mL ofloxacin and 51 µg/mL SPI009. After 5 hours of treatment, bacterial cultures were diluted 1:100 into fresh medium and grown for 40 hours at 37°C, shaking at 200 rpm. At timepoints 20, 24 and 40 hours, optical density was measured using a Synergy MX multimode reader (Biotek). Average OD_{595} was calculated for each 96-well plate and used to correct mutant OD_{595} values. Mutants were identified as sensitive if the OD_{595} after 24 hours of growth was ≤ 0.3 x average OD_{595} after 24 hours. Alternatively, mutants having an OD_{595} > 3 x average were defined as resistant. To allow identification of SPI009 specific effects, selected mutants showing a clear sensitivity or

resistance for ofloxacin were excluded, after which functional enrichment analysis based on PseudoCAP classifications was done for all selected mutants.

Figure S2

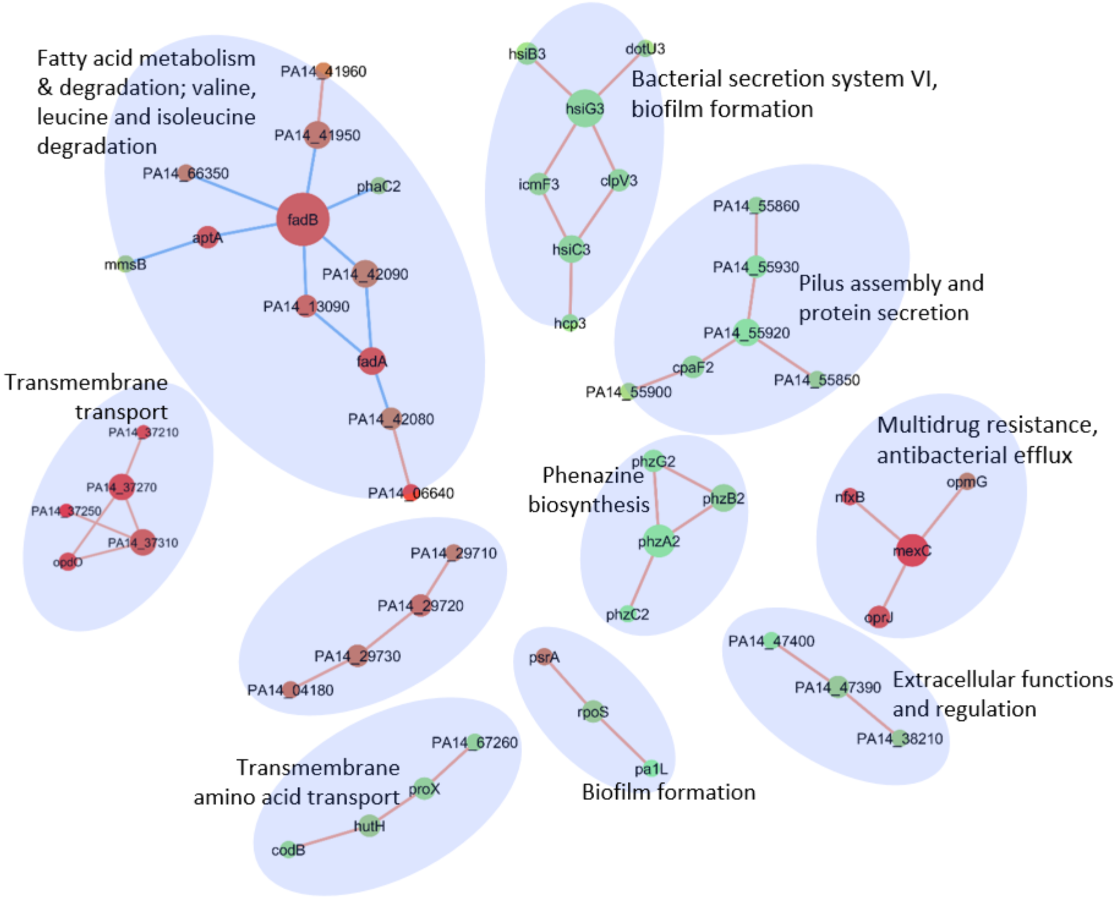

Figure S2: PheNetic network analysis of differential expression data upon treatment with SPI009. PheNetic network analysis was performed on obtained RNAseq data using PheNetic (downstream, cost = 0,25) and the custom made UCBBP-PA14 interaction network. Sub-networks of only 2 genes were excluded from analysis. Nodes and connecting lines represent genes and interactions differentially active upon treatment with 50 µM SPI009. Red and green circles represent up- and downregulated genes with color intensity correlated to the absolute log-fold change. Yellow and blue lines represent protein-protein and metabolic interactions, respectively.

Figure S3

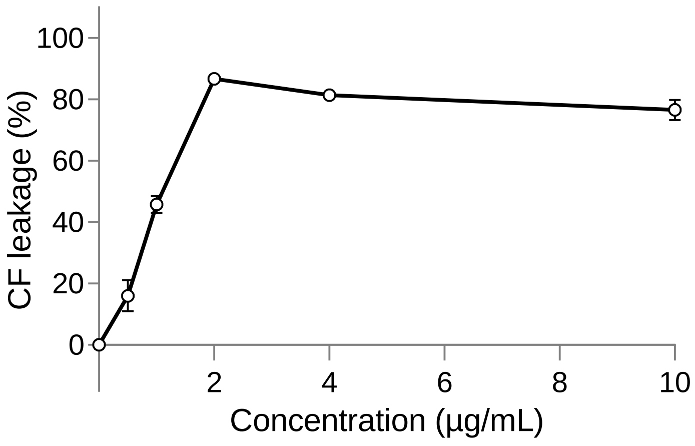

Figure S3: Disruption of artificial bacterial membranes by polymyxin B. SUV's were treated for 15 minutes with increasing concentrations of polymyxin B. % CF leakage, as determined relative to the positive control (0.5% Triton X-100) is represented on the y-axis. Data points represent the mean of three independent experiments ± SEM.

Figure S4

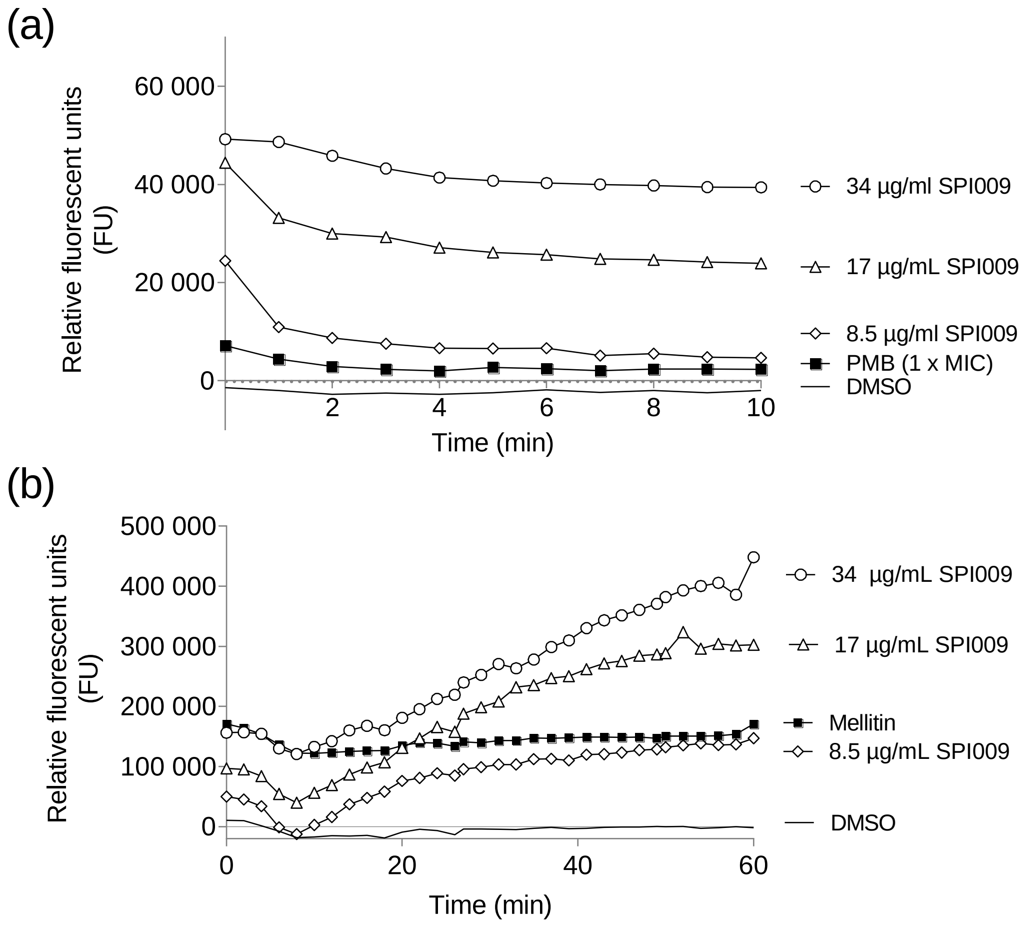

Figure S4: Time kinetics of outer and inner membrane damage. (a) NPN fluorescence was measured every minute for 10 minutes to assess membrane permeabilization kinetics following treatment with a positive control (PMB, filled square), DMSO (black line), 8.5 µg/mL SPI009 (open diamonds), 17 µg/mL SPI009 (open triangles) or 34 µg/mL (open circles). Results are the mean of at least 4 independent experiments. (b) SG fluorescence was measured every minute during 60 minutes to assess membrane permeabilization kinetics following treatment with a positive control (mellitin, filled square), DMSO (black line), 8.5 µg/mL SPI009 (open diamonds), 17 µg/mL SPI009 (open triangles) or 34 µg/mL (open circles). Results are the mean of at least 4 independent experiments.

Figure S5

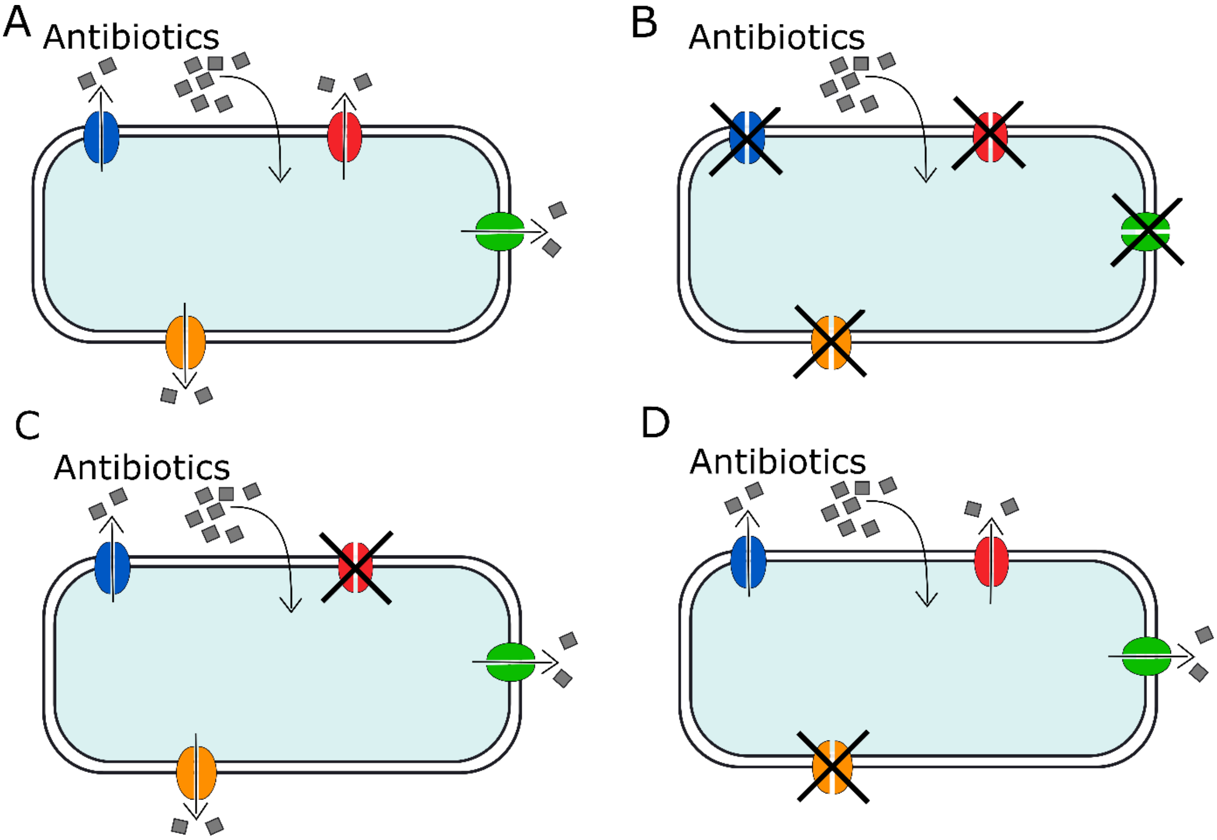

Figure S5: Visual representation of the *P. aeruginosa* efflux mutants discussed in section 3.7. (a) The *P. aeruginosa* YM WT strain contains all major RND efflux pumps; MexAB-OprM (blue), MexCD-OprJ (red), MexEF-OprN (green) and MexXY-OprM (orange), resulting in efficient efflux. **(b)** Deletion of all four Mex efflux pumps, as is the case for *P. aeruginosa* YM64 will strongly decrease antibiotic efflux. **(c)** and **(d)** represent single deletion mutants of, respectively, *mexCD-oprJ* and *mexXY*, resulting in inactivation of the respective efflux pumps.

Figure S6

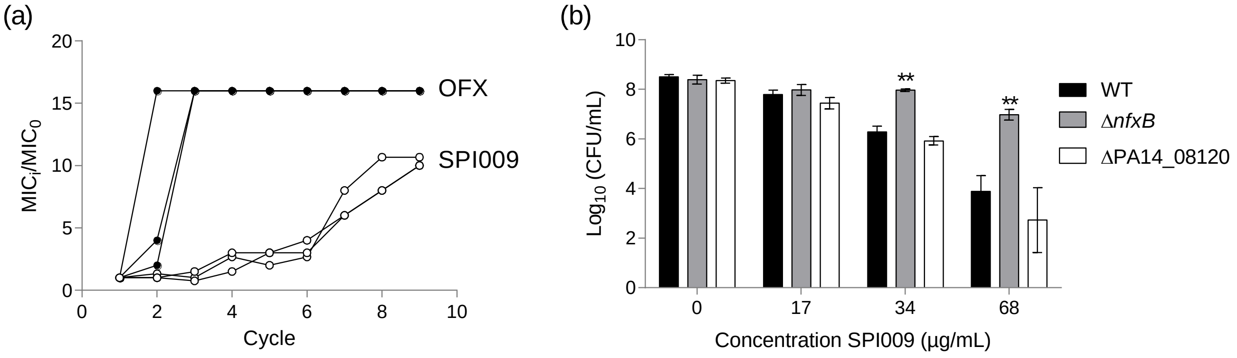

Figure S6: High selection pressure resistance development in SPI009. a) 6 independent PA14WT strains were treated daily with, if necessary, increasing concentrations of ofloxacin (filled circles) or SPI009 (open circles) in a MIC-based evolution experiment. The y-axis represents the fold increase in MIC-value relative to the MIC at the start of the experiment. Three separate strains are shown for each antimicrobial. b) Stationary phase cultures of PA14WT (black bars) and transposon mutants of *nfxB* (grey bars) and PA14_08120 (white bars), the two SNP's identified in all SPI009 resistant strains, were treated for 5 hours with increasing concentrations of SPI009. Data points represent the average of at least three independent repeats ± SEM. Statistical differences between the WT and mutants were determined by means of multiple t tests using the Holm-Sidak correction for multiple comparisons. ** $P < 0.01$.

Table S1: Functional enrichment analysis of *P. aeruginosa* transposon mutants showing increased sensitivity towards SPI009.

PseudoCAP classes showing a functional enrichment, as determined via Fisher's exact test, are highlighted in grey.

Functional class	# liberati mutants in class	# sensitive mutants	Fisher's exact test P-value	Gene list
**2-component systems**	139	4	0.547598326	*phoP, algR,* PA14_27800, PA14_11630
**Amino acid biosynthesis and metabolism**	235	9	0.102488053	*argR, carB, thrC, argH, hisH, aguA, hutH,* PA14_05480, PA14_63120
**Adaptation and protection**	132	7	0.024742372	*lon, dksA, katA, bfrB, ppk, pa1L,* PA14_57840
**Antibiotic resistance and protection**	60	1	1	*mexD*
**Carbon compound catabolism**	144	5	0.245592847	*msuD, pa1L, pcaG, pykA, treA*
**Cell wall/LPS/capsule**	122	7	0.016787118	*comL, lpxC, pa1L, wzz, arnT, wbpW,* PA14_66200, PA14_08540
**Central intermediary metabolism**	112	4	0.310421356	*tpiA, nasA, hutH,* PA14_04150
**DAN replication, modification and repair**	97	3	0.471023797	*phr, dksA, sbcB*
**Energy metabolism**	217	8	0.146264308	*nuoJ, lldD, tpiA, ccpR, pykA,* PA14_69110, PA14_42120, PA14_44360
**Export apparatus**	102	5	0.071430469	*pscL, xcpT, xcpR,* PA14_40170, PA14_66580
**Fatty acid and phospholipid metabolism**	157	1	0.264525635	PA14_38640
**Membrane proteins**	456	14	0.17871873	*shaB, dnpA, nasA,* PA14_38320, PA14_36170, PA14_40170, PA14_63970, PA14_45070, PA14_52350, PA14_65030, PA14_68830
**Motility and attachment**	112	2	1	*cupC1*, PA14_61530
**Nucleotide biosynthesis & metabolism**	62	3	0.152827949	*carB, ppk, PA14_05790*
**Putative enzymes**	432	11	0.604476392	*nagZ*, PA14_35880, PA14_69110, PA14_62420, PA14_49070, PA14_04150, PA14_16620, PA14_67440, PA14_07780, PA14_53420, PA14_40030
**Related to phage, transposon, plasmid**	67	4	0.057868633	PA14_61840, PA14_67810, PA14_48880, PA14_53570
**Secreted factors**	79	1	1	*rhlB*
**Synthesis of co-factors**	139	3	1	*mvfR, thrC,* PA14_02420

**Transcription, RNA processing and degradation**	44	2	0.249555074	*rho, pcnB*
**Transcriptional regulators**	357	9	0.575666494	*phoP, algR, argR, dksA, mvfR, PA14_06400, PA14_68920, PA14_51340, PA14_56430*
**Translation, post-translational modification, degradation**	146	6	0.136993652	*selD, pheT, nusA, lon,*PA14_67810, PA14_70480
**Transport small molecules**	600	19	0.100567331	*mexA, sppB,bfrB, shaB, znuC, nasA, gabP, corA, fepG,* PA14_69070, PA14_47330, PA14_19540, PA14_38320, PA14_69070, PA14_57870, PA14_40170, PA14_45070, PA14_47560, PA14_30590
**Hypothetical**	1557	36	0.681128806	*dnpA, argH, wzz,pykA,* PA14_62420, PA14_47330, PA14_42160, PA14_36170, PA14_07240, PA14_57840, PA14_16620, PA14_21490, PA14_08490, PA14_14430, PA14_08330, PA14_63970, PA14_63970, PA14_67440, PA14_07780, PA14_07780, PA14_53100, PA14_51570, PA14_59070 PA14_03370, PA14_15140, PA14_42160, PA14_52350, PA14_70480, PA14_07360, PA14_69010, PA14_24570, PA14_66580, PA14_32750, PA14_40030, PA14_21480, PA14_61010, PA14_03250, PA14_08540

Table S2: Differentially expressed genes in *P. aeruginosa* upon treatment with SPI009. RNA sequence analysis revealed a set of 297 genes showing a differential up- or down-regulation specifically for SPI009 and with log_2-fold changes >1.

Gene name	Corresponding PAO1 locus	Product name and/or function	Fold change (Log_2)
PA14_26850	PA2880	hypothetical protein	3.64
gpo	PA2826	glutathione peroxidase	3.38
PA14_37250	PA2114	major facilitator transporter	3.25
gcvH2	PA2446	glycine cleavage system protein H	2.99
mexC	PA4599	multidrug efflux RND membrane fusion protein	2.91
PA14_37210	PA2116	hypothetical protein	2.70
PA14_06640	PA0508	acyl-CoA dehydrogenase	2.70
PA14_27530	PA2825	MarR family transcriptional regulator	2.66
PA14_37270	PA2112	LamB/YcsF family protein	2.65
opdO	PA2113	porin	2.63
nfxB (PA14_60810)	PA4600	transcriptional regulator NfxB	2.54
PA14_44230	/	hypothetical protein	2.51
gcvP2	PA2445	glycine dehydrogenase	2.46
PA14_06600	PA0506	acyl-CoA dehydrogenase	2.43
oprJ	PA4597	outer membrane protein OprJ	2.39
fadA	PA3013	3-ketoacyl-CoA thiolase	2.33
aptA	PA0132	beta alanine--pyruvate transaminase	2.27
PA14_32950	PA2448	hypothetical protein	2.25
htpX	PA2830	heat shock protein HtpX	2.24
fadB	PA3014	multifunctional fatty acid oxidation complex subunit α	2.14
PA14_47640	PA1282	major facilitator transporter	2.08
PA14_37310	PA2110	hypothetical protein	2.08
PA14_71900	PA5446	hypothetical protein	2.05
PA14_36020	/	paraquat-inducible protein B	2.03
nfxB (PA14_60860)	PA4596	transcriptional regulator NfxB	1.96
PA14_16640	PA3691	lipoprotein	1.93
PA14_64520	PA4880	bacterioferritin	1.90
PA14_32470	PA2487	hypothetical protein	1.87
PA14_13090	PA3925	acyl-CoA thiolase	1.83
PA14_62390	PA4714	hypothetical protein	1.82
PA14_25840	PA2953	electron transfer flavoprotein-ubiquinone oxidoreductase	1.80
PA14_25840	PA0840	oxidoreductase	1.77
PA14_36030	/	paraquat-inducible protein A	1.74
PA14_68840	PA5212	hypothetical protein	1.73
glyA2	PA2444	serine hydroxymethyltransferase	1.73
aspA	PA5429	aspartate ammonia-lyase	1.71
PA14_44311	PA1559	hypothetical protein	1.61
PA14_24360	/	hypothetical protein	1.61
PA14_47120	PA1324	hypothetical protein	1.60

PA14_29720	PA2658	hypothetical protein	1.60
PA14_00620	PA0049	hypothetical protein	1.53
pfpI	PA0355	protease PfpI	1.52
oprH	PA1178	PhoP/Q and low Mg^{2+} inducible outer membrane protein	1.50
PA14_72930	PA5528	hypothetical protein	1.49
PA14_53410	PA0839	transcriptional regulator	1.49
osmC	PA0059	osmotically inducible protein OsmC	1.48
deaD	PA2840	ATP-dependent RNA helicase	1.45
PA14_40890	PA1828	short chain dehydrogenase	1.45
PA14_04180	PA0320	hypothetical protein	1.40
PA14_02060	PA0165	hypothetical protein	1.39
*prmF*or *arnC*	PA3553	glycosyl transferase family protein	1.39
PA14_41950	PA1748	enoyl-CoA hydratase	1.37
PA14_40880	PA1829	hypothetical protein	1.37
rplJ	PA4272	50S ribosomal protein L10	1.35
PA14_22730	PA3206	two-component sensor	1.30
pstC	PA5368	membrane protein component of ABC phosphate transporter	1.29
etfB	PA2952	electron transfer flavoprotein subunit beta	1.29
pstA	PA5367	phosphate ABC transporter permease	1.28
arnB	PA3552	UDP-4-amino-4-deoxy-L-arabinose--oxoglutarate aminotransferase	1.28
yccA	PA2604	hypothetical protein	1.27
PA14_29730	PA2657	two-component response regulator	1.27
aceA	PA2634	isocitrate lyase	1.27
PA14_04710	/	hypothetical protein	1.27
PA14_47130	PA1323	hypothetical protein	1.27
ohr	PA2850	organic hydroperoxide resistance protein	1.26
osmE	PA4876	DNA-binding transcriptional activator OsmE	1.25
PA14_33160	PA2433	hypothetical protein	1.24
rplA	PA4273	50S ribosomal protein L1	1.24
yegH	PA1331	TerC family protein	1.24
PA14_53500	PA0833	hypothetical protein	1.23
PA14_31870	PA2528	RND efflux membrane fusion protein	1.23
PA14_57610	/	/	1.21
typA	PA5117	GTP-binding protein TypA	1.19
psrA	PA3006	transcriptional regulator PsrA	1.19
nuoI	PA2644	NADH dehydrogenase subunit I	1.19
tsf	PA3655	elongation factor Ts	1.18
rplL	PA4271	50S ribosomal protein L7/L12	1.17
PA14_05040	PA0386	coproporphyrinogen III oxidase	1.17
PA14_29710	PA2659	hypothetical protein	1.16
PA14_68110	PA5157	transcriptional regulator	1.16
rplX	PA4252	50S ribosomal protein L24	1.16
nuoD	PA2639	bifunctional NADH:ubiquinone oxidoreductase subunit C/D	1.16
PA14_66350	PA5020	acyl-CoA dehydrogenase	1.15

PA14_00570	PA0045	lipoprotein	1.15
emrA	PA5159	multidrug resistance protein	1.15
rplN	PA4253	50S ribosomal protein L14	1.14
rplE	PA4251	50S ribosomal protein L5	1.14
rpsA	PA3162	30S ribosomal protein S1	1.13
PA14_37200	PA2117	hypothetical protein	1.13
ordL	PA2776	hypothetical protein	1.13
rplK	PA4274	50S ribosomal protein L11	1.13
*fmt*or *arnA*	PA3554	bifunctional UDP-glucuronic acid decarboxylase/UDP-4-amino-4-deoxy-L-arabinose formyltransferase	1.13
mocA	PA3795	oxidoreductase	1.13
rplI	PA4932	50S ribosomal protein L9	1.12
PA14_16370	PA3712	hypothetical protein	1.12
rplW	PA4261	50S ribosomal protein L23	1.12
rplR	PA4247	50S ribosomal protein L18	1.12
PA14_64490	PA4877	hypothetical protein	1.12
mdr	PA3136	secretion protein	1.12
ybiT	PA1964	ABC transporter ATP-binding protein	1.11
etfA	PA2951	electron transfer flavoprotein subunit alpha	1.11
rpsQ	PA4254	30S ribosomal protein S17	1.11
rpsB	PA3656	30S ribosomal protein S2	1.10
PA14_04270	PA0327	transcriptional regulator	1.10
PA14_42090	PA1736	acetyl-CoA acetyltransferase	1.09
PA14_41960	PA1747	hypothetical protein	1.08
ycaL	PA0277	Zn-dependent protease with chaperone function	1.08
fadH1	PA3092	2,4-dienoyl-CoA reductase	1.07
PA14_40860	PA1830	hypothetical protein	1.07
rpmC	PA4255	50S ribosomal protein L29	1.07
rplP	PA4256	50S ribosomal protein L16	1.07
potA	PA3607	polyamine transport protein PotA	1.06
PA14_54720	/	hypothetical protein	1.06
galU	PA2023	UTP-glucose-1-phosphate uridylyltransferase	1.06
ycfJ	PA3819	hypothetical protein	1.06
PA14_69270	PA5246	hypothetical protein	1.05
rplD	PA4262	50S ribosomal protein L4	1.05
PA14_41690	PA1768	hypothetical protein	1.05
PA14_23520	PA3137	MFS transporter	1.04
glnA	PA5119	glutamine synthetase	1.04
emrB	PA5160	drug efflux transporter	1.04
opmG	PA5158	outer membrane protein	1.03
gpmA	PA1831	hypothetical protein	1.03
pitA	PA4292	phosphate transporter	1.03
PA14_07550	PA0578	hypothetical protein	1.02
rplB	PA4260	50S ribosomal protein L2	1.02
rplV	PA4258	50S ribosomal protein L22	1.02
PA14_21640	PA3277	short chain dehydrogenase	1.02
PA14_42080	PA1737	3-hydroxyacyl-CoA dehydrogenase	1.02

rpsN	PA4250	30S ribosomal protein S14	1.02
rpsS	PA4259	30S ribosomal protein S19	1.01
rpsE	PA4246	30S ribosomal protein S5	1.00
ugd	PA2022	nucleotide sugar dehydrogenase	1.00
PA14_56700	PA4360	hypothetical protein	1.00
PA14_41710	PA1767	hypothetical protein	1.00
rplF	PA4248	50S ribosomal protein L6	1.00
rpsC	PA4257	30S ribosomal protein S3	1.00
PA14_32490	PA2485	hypothetical protein	1.00
PA14_22130	/	hypothetical protein	-1.00
PA14_68280	PA5168	dicarboxylate transporter	-1.00
PA14_37745	PA2069	carbamoyl transferase	-1.01
orfH	/	UDP-N-acetyl-D-mannosaminuronate dehydrogenase	-1.01
qscR	PA1898	transcriptional regulator	-1.01
PA14_37760	PA2068	MFS transporter	-1.02
PA14_70780	PA5363	hypothetical protein	-1.02
PA14_65090	PA4929	hypothetical protein	-1.02
proW	PA5095	ABC transporter permease	-1.02
mmsB	PA3569	3-hydroxyisobutyrate dehydrogenase	-1.02
PA14_54870	/	hypothetical protein	-1.02
PA14_49330	PA1166	hypothetical protein	-1.03
PA14_38190	PA2036	hypothetical protein	-1.03
coIII	PA0108	cytochrome c oxidase subunit III	-1.03
PA14_06130	PA0468	hypothetical protein	-1.04
PA14_42150	PA1731	hypothetical protein	-1.04
PA14_01780	PA0144	nucleoside 2-deoxyribosyltransferase	-1.04
PA14_58360	PA4497	ABC transporter substrate-binding protein	-1.04
napE	PA1177	periplasmic nitrate reductase NapE	-1.04
lasB	PA3724	elastase LasB	-1.05
arcD	PA5170	arginine/ornithine antiporter	-1.05
fepC	PA4158	ferric enterobactin transport protein FepC	-1.05
clpA	PA0459	ClpA/B protease ATP binding subunit	-1.05
PA14_55200	PA0704	amidase	-1.05
PA14_12680	PA3957	short chain dehydrogenase	-1.05
PA14_35700	/	hypothetical protein	-1.06
PA14_48530	PA1221	AMP-binding protein	-1.06
lpdV	PA2250	dihydrolipoamide dehydrogenase	-1.06
PA14_32630	PA2475	cytochrome P450	-1.07
phaD	PA5059	TetR family transcriptional regulator	-1.07
PA14_46550	/	ribonuclease	-1.07
PA14_46540	/	hypothetical protein	-1.07
phaC2	PA5058	poly(3-hydroxyalkanoic acid) synthase 2	-1.07
PA14_48600	PA1215	AMP-binding protein	-1.08
PA14_21190	PA3311	hypothetical protein	-1.08
exbB1	PA0198	transport protein ExbB	-1.08
ynfM	PA5030	MFS transporter	-1.08

PA14_21510	PA3289	hypothetical protein	-1.09
hslR	PA5195	heat shock protein	-1.09
hutT	PA5097	amino acid permease	-1.09
PA14_08060	PA0621	tail fiber assembly protein	-1.10
PA14_67370	PA5101	hypothetical protein	-1.10
phnC	PA3384	ABC phosphonate transporter ATP-binding protein	-1.10
PA14_36860	/	hypothetical protein	-1.10
tauD	PA3935	taurine dioxygenase	-1.11
PA14_73090	PA5542	hypothetical protein	-1.11
PA14_35710	/	hypothetical protein	-1.12
PA14_07020	PA0540	hypothetical protein	-1.12
PA14_38210	PA2034	hypothetical protein	-1.12
PA14_12910	PA3939	hypothetical protein	-1.13
PA14_04330	PA0332	hypothetical protein	-1.14
PA14_50880	PA1041	hypothetical protein	-1.14
PA14_54010	PA0791	transcriptional regulator	-1.14
PA14_62250	PA4703	hypothetical protein	-1.15
PA14_19480	PA3451	hypothetical protein	-1.15
PA14_32280	PA2504	hypothetical protein	-1.15
PA14_28190	/	tRNA-Val	-1.16
PA14_00080	PA0007	hypothetical protein	-1.16
PA14_36470	PA2174	hypothetical protein	-1.16
PA14_51600	PA0981	hypothetical protein	-1.16
pyoS3I	/	immunity protein S3I structureal gene	-1.16
PA14_46780	PA1353	hypothetical protein	-1.16
PA14_11140	PA4078	nonribosomal peptide synthetase	-1.17
PA14_61500	PA4648	hypothetical protein	-1.17
rpoS	PA3622	RNA polymerase sigma factor RpoS	-1.18
PA14_64920	PA4915	methyl-accepting chemotaxis protein	-1.18
PA14_48560	PA1218	hypothetical protein	-1.18
cpg2	PA2787	glutamate carboxypeptidase	-1.19
PA14_10360	PA4141	hypothetical protein	-1.19
PA14_47390	PA1301	transmembrane sensor	-1.19
PA14_13920	/	hypothetical protein	-1.19
PA14_20000	PA3409	transmembrane sensor	-1.20
hupA	PA5348	HU family DNA-binding protein	-1.20
PA14_10770	PA4112	sensor/response regulator hybrid	-1.20
PA14_54240	PA0776	hypothetical protein	-1.21
PA14_55780	PA4293	two-component sensor	-1.21
PA14_23400	/	hypothetical protein	-1.21
nlpD	PA3623	hypothetical protein	-1.21
PA14_55900	PA4303	hypothetical protein	-1.22
fdhD	PA5180	formate dehydrogenase accessory protein FdhD	-1.22
PA14_60960	PA4607	hypothetical protein	-1.22
PA14_65040	PA4925	hypothetical protein	-1.23
PA14_13110	PA3924	long-chain-fatty-acid--CoA ligase	-1.23
PA14_12940	PA3937	taurine ABC transporter ATP-binding protein	-1.23

PA14_55850	PA4299	pilus assembly protein	-1.24
PA14_68440	PA5181	oxidoreductase	-1.25
PA14_58740	/	hypothetical protein	-1.25
orfE	/	polysaccharide biosynthesis protein	-1.26
PA14_64930	PA4916	hypothetical protein	-1.26
fptB	PA4220	hypothetical protein	-1.26
PA14_39270	PA1951	hypothetical protein	-1.28
dotU3	PA2362	hypothetical protein	-1.29
coxG	PA0107	cytochrome C oxidase assembly protein	-1.29
PA14_61940	PA4682	hypothetical protein	-1.30
PA14_49410	PA1159	cold-shock protein	-1.30
ssuB	PA3442	aliphatic sulfonates transport ATP-binding subunit	-1.31
PA14_15280	PA3772	hypothetical protein	-1.32
PA14_38220	PA2033	hypothetical protein	-1.32
PA14_61920	PA4681	hypothetical protein	-1.32
PA14_28790	/	hypothetical protein	-1.33
PA14_37690	PA2072	sensory box protein	-1.33
PA14_34100	PA2363	hypothetical protein	-1.35
bfd	PA3530	hypothetical protein	-1.35
PA14_21480	PA3291	hypothetical protein	-1.36
PA14_29330	/	hypothetical protein	-1.37
PA14_48620	PA1213	clavaminic acid synthetase	-1.37
PA14_34080	PA2364	hypothetical protein	-1.38
pmtA	PA0798	phospholipid methyltransferase	-1.40
PA14_40240	PA1876	ABC transporter ATP-binding protein/permease	-1.41
PA14_40260	PA1874	hypothetical protein	-1.42
PA14_59230	PA0984	colicin immunity protein	-1.42
PA14_39860	PA1907	hypothetical protein	-1.42
PA14_40740	/	hypothetical protein	-1.42
pqqA	PA1985	coenzyme PQQ synthesis protein PqqA	-1.42
PA14_55790	PA4294	hypothetical protein	-1.42
rhlC	PA1130	rhamnosyltransferase 2	-1.43
icmF3	PA2361	hypothetical protein	-1.44
opdB	PA2700	porin	-1.44
phzE1	PA4214	phenazine biosynthesis protein PhzE	-1.44
PA14_46530	/	hypothetical protein	-1.45
proX	PA5096	ABC transporter substrate-binding protein	-1.46
phzB2	PA1900	phenazine biosynthesis protein	-1.46
PA14_27990	PA2794	sialidase	-1.46
PA14_01330	PA0109	hypothetical protein	-1.46
PA14_67260	PA5093	histidine/phenylalanine ammonia-lyase	-1.46
apaH	PA1906	hydrolase	-1.47
PA14_55860	PA4300	hypothetical protein	-1.48
hsiB3	PA2365	hypothetical protein	-1.49
trbF	/	conjugal transfer protein TrbF	-1.50
ssuF	PA3441	molybdopterin-binding protein	-1.51

PA14_12260	PA3986	hypothetical protein	-1.52
PA14_13210	/	hypothetical protein	-1.55
codB	PA5099	cytosine/purines uracil thiamine allantoin permease	-1.55
cpaF2	PA4302	type II secretion system protein	-1.55
PA14_26020	PA2939	aminopeptidase	-1.56
PA14_61010	PA4611	hypothetical protein	-1.57
clpV3	PA2371	ClpA/B-type protease	-1.60
hcp3	PA2367	hypothetical protein	-1.60
PA14_37670	/	hypothetical protein	-1.63
hsiC3	PA2366	hypothetical protein	-1.64
rmf	PA3049	ribosome modulation factor	-1.65
hsiG3	PA2369	hypothetical protein	-1.67
PA14_58040	PA4471	hypothetical protein	-1.67
hvn	PA1914	hypothetical protein	-1.73
PA14_40750	/	hypothetical protein	-1.79
PA14_55880	PA4301	hypothetical protein	-1.81
PA14_28780	/	hypothetical protein	-1.83
PA14_55930	PA4305	pilus assembly protein	-1.85
phzG2	PA1905	pyridoxamine 5'-phosphate oxidase	-1.85
PA14_47400	PA1300	RNA polymerase ECF-subfamily sigma-70 factor	-1.89
PA14_55920	PA4304	type II secretion system protein	-1.93
phzA2	PA1899	phenazine biosynthesis protein	-1.99
PA14_60480	PA4570	hypothetical protein	-1.99
phzC2	PA1901	phenazine biosynthesis protein PhzC	-2.00
PA14_33930	PA2375	hypothetical protein	-2.05
yeiH	PA5383	hypothetical protein	-2.12
PA14_55940	PA4306	hypothetical protein	-2.18
pa1L	PA2570	PA-I galactophilic lectin	-2.19
PA14_61380	/	hypothetical protein	-2.98
